# Genomic Properties and Temporal Analysis of the Interaction of an Invasive *Escherichia albertii* With Epithelial Cells

**DOI:** 10.3389/fcimb.2020.571088

**Published:** 2020-12-16

**Authors:** Fabiano T. Romão, Fernando H. Martins, Rodrigo T. Hernandes, Tadasuke Ooka, Fernanda F. Santos, Denise Yamamoto, Alexis Bonfim-Melo, Nina Jones, Tetsuya Hayashi, Waldir P. Elias, Vanessa Sperandio, Tânia A. T. Gomes

**Affiliations:** ^1^ Departamento de Microbiologia, Imunologia e Parasitologia, Escola Paulista de Medicina, Universidade Federal de São Paulo (EPM-UNIFESP), São Paulo, Brazil; ^2^ Department of Microbiology, University of Texas Southwestern Medical Center, Dallas, TX, United States; ^3^ Department of Biochemistry, University of Texas Southwestern Medical Center, Dallas, TX, United States; ^4^ Laboratório de Bacteriologia, Instituto Butantan, São Paulo, Brazil; ^5^ Instituto de Biociências, Universidade Estadual Paulista (UNESP), Botucatu, Brazil; ^6^ Department of Microbiology, Graduate School of Medical and Dental Sciences, Kagoshima University, Kagoshima, Japan; ^7^ Universidade Santo Amaro, São Paulo, Brazil; ^8^ Department of Molecular and Cellular Biology, University of Guelph, Guelph, ON, Canada; ^9^ Department of Bacteriology, Faculty of Medical Sciences, Kyushu University, Fukuoka, Japan

**Keywords:** *Escherichia albertii*, attaching and effacing lesion, type three secretion system, invasion, pathogenicity, diarrhea, Tir cytoskeleton-coupling protein/EspFu, locus of enterocyte effacement

## Abstract

Diarrhea is one of the main causes of infant mortality worldwide, mainly in the developing world. Among the various etiologic agents, *Escherichia albertii* is emerging as an important human enteropathogen. *E. albertii* promote attaching and effacing (AE) lesions due to the presence of the locus of enterocyte effacement (LEE) that encodes a type three secretion system (T3SS), the afimbrial adhesin intimin and its translocated receptor, Tir, and several effector proteins. We previously showed that *E. albertii* strain 1551-2 invades several epithelial cell lineages by a process that is dependent on the intimin-Tir interaction. To understand the contribution of T3SS-dependent effectors present in *E. albertii* 1551-2 during the invasion process, we performed a genetic analysis of the LEE and non-LEE genes and evaluated the expression of the LEE operons in various stages of bacterial interaction with differentiated intestinal Caco-2 cells. The kinetics of the ability of the 1551-2 strain to colonize and form AE lesions was also investigated in epithelial HeLa cells. We showed that the LEE expression was constant during the early stages of infection but increased at least 4-fold during bacterial persistence in the intracellular compartment. An *in silico* analysis indicated the presence of a new *tccP/espF_U_* subtype, named *tccP3*. We found that the encoded protein colocalizes with Tir and polymerized F-actin during the infection process *in vitro*. Moreover, assays performed with Nck null cells demonstrated that the 1551-2 strain can trigger F-actin polymerization in an Nck-independent pathway, despite the fact that TccP3 is not required for this phenotype. Our study highlights the importance of the T3SS during the invasion process and for the maintenance of *E. albertii* 1551-2 inside the cells. In addition, this work may help to elucidate the versatility of the T3SS for AE pathogens, which are usually considered extracellular and rarely reach the intracellular environment.

## Introduction

Diarrhea is one of the leading causes of infant mortality worldwide, mainly in the developing world. Many enteropathogens are associated with this disease (Kotloff et al., 2013), including *Escherichia albertii*, which is emerging as an important enteropathogen of humans and birds (Huys et al., 2003; Oaks et al., 2010; Gomes et al., 2020).

Similar to enteropathogenic and enterohemorrhagic *E. coli* (EPEC and EHEC), *E. albertii* strains harbor a pathogenicity island (PAI) called the locus of enterocyte effacement (LEE) (Hyma et al., 2005; Ooka et al., 2015; Gomes et al., 2020). The LEE contains five polycistronic operons (*LEE1* to *LEE5*), two bicistronic operons and four individual transcriptional units (McDaniel et al., 1995; Elliott et al., 1998; Gaytan et al., 2016), and it encodes a type three secretion system (T3SS), which is a molecular machinery that injects several effector proteins into the host cell cytosol (Jarvis et al., 1995).

The *ler* gene, located in the LEE, encodes a transcriptional regulator, which positively regulates many EPEC virulence factor-encoding genes in the LEE region (Mellies et al., 1999), except for genes within the *LEE1* operon (Berdichevsky et al., 2005). The Ler protein counteracts silencing by the H-NS global repressor, thus promoting the expression of the LEE genes (Mellies et al., 1999; Bustamante et al., 2001). The *LEE1, LEE2*, and *LEE3* operons encode most of the structural components of the T3SS (Elliott et al., 1998), while *LEE4* contains genes encoding the needle and the translocon proteins (EspA, EspB, and EspD) (Knutton et al., 1998; Ide et al., 2001). *LEE5* contains the *eae* and *tir* genes, which encode the adhesin intimin and its translocated receptor, Tir, respectively (Jerse et al., 1990; Kenny et al., 1997). The interaction between Tir and intimin leads to reorganization of the host cell cytoskeleton, with effacement of the enterocyte microvilli and F-actin accumulation underneath the adhering bacteria, forming a pedestal-like structure. These alterations are referred to as attaching and effacing (AE) lesions (Moon et al., 1983; Knutton et al., 1989). Besides the LEE effectors, various T3SS-dependent non-LEE (Nle)-encoded effector genes have been described (Deng et al., 2004; Tobe et al., 2006; Wong et al., 2011; Serapio-Palacios and Finlay, 2020). Nle proteins have been shown to disturb the host cell cytoskeleton and tight junctions as well as to modulate the host inflammatory response (Dean and Kenny, 2009; Wong et al., 2011; Pearson et al., 2016). *E. albertii* strains also contain multiple non-LEE effectors (Ooka et al., 2015).

AE pathogens can use two distinct pathways to trigger F-actin for pedestal formation: Tir-Nck dependent and/or Tir-Nck independent. In the Tir-Nck dependent pathway, tyrosine residue 474 (Y_474_) of the Tir receptor is phosphorylated by host cell kinase(s), which recruits Nck to initiate localized actin assembly (Kenny, 1999). On the other hand, the Nck-independent pathway uses a non-LEE effector termed EspF_U_/TccP (Tir cytoskeleton-coupling protein) that links to the C terminus of Tir (EPEC NPY_454_/O157:H7 EHEC NPY_458_) *via* the host adaptor IRTKS/IRSp53. Both pathways activate N-WASP, which recruits and activates the Arp2/3 complex, culminating in actin polymerization (Campellone et al., 2004; Garmendia et al., 2004; Vingadassalom et al., 2009).

Most AE bacteria are extracellular pathogens, but several studies have shown that some of them can be invasive (Hernandes et al., 2008; Sampaio et al., 2009; Yamamoto et al., 2009; Pacheco et al., 2014). We have previously shown that *E. albertii* strain 1551-2, formerly classified as atypical EPEC (Vieira et al., 2001; Hernandes et al., 2006), is able to invade several epithelial cell lineages such as HeLa cells and differentiated intestinal T84 and Caco-2 cells (Hernandes et al., 2008; Yamamoto et al., 2009; Pacheco et al., 2014; Yamamoto et al., 2017). Moreover, we showed that the invasion process of this strain is dependent on the intimin-Tir interaction, since a coisogenic *eae* mutant was no longer invasive, even though its ability to adhere to epithelial cells was unaltered (Hernandes et al., 2008; Yamamoto et al., 2017). Furthermore, the 1551-2 strain persisted within Caco-2 cells for 24 h (Pacheco et al., 2014). However, further research is necessary to understand the steps for this colonization process, including analysis of effector protein functions and the expression of the LEE genes during this process.

To unravel the pathogenesis of the *E. albertii* 1551-2 strain, here we performed a genetic analysis of the LEE and non-LEE genes and evaluated the expression of the LEE operons in various stages of the interaction of this strain with differentiated intestinal Caco-2 cells. The ability of 1551-2 strain to colonize and form pedestals was also investigated in epithelial HeLa cells in detail.

## Material and Methods

### Bacterial Strain

The *E. albertii* 1551-2 strain, which was formerly classified as atypical EPEC, was isolated from a child (23 months old) with diarrhea, in the absence of other recognized pathogens, during an epidemiological study on diarrhea, which was carried out in 1989 at the Universidade Federal de São Paulo (UNIFESP), Brazil (Vieira et al., 2001). The strain was routinely grown aerobically in Lysogeny Broth (LB) medium or Dulbecco’s modified Eagle’s medium (DMEM, Gibco, USA) at 37°C. As this strain is nalidixic acid resistant, it was cultured in the presence of this antibiotic (20 µg/ml), when necessary, and stocked in LB supplemented with 15% glycerol at -80°C.

### Sequence Analyses of Non-LEE T3SS-Dependent Effectors

The protein-coding genes of strain 1551-2 (accession number: CP025317.1, Romão et al., 2018) were analyzed with BLASTp to investigate the non-LEE T3SS-dependent effector repertoire using the non-LEE T3SS effectors that have been identified in the enterohemorrhagic *E. coli* (EHEC) O157 strain Sakai (Hayashi et al., 2001), the EPEC O127:H6 strain E2348/69 (Iguchi et al., 2009), and the EPEC O111:H^-^ strain B171 (Ogura et al., 2008) as queries, with filtering by hit length coverage (>70%) and sequence identity (>30%).

### DNA and Genetic Manipulations

Plasmid pKC471 is a derivative of the low copy number vector pK187 (kan^R^) that harbors the *tccP* gene from the O157 EHEC EDL933 strain cloned upstream of a myc epitope (Campellone et al., 2004). To construct pTccP3, a 564 bp DNA fragment (from 93 bp upstream of the ATG start codon until just before the *tccP3* ORF stop codon), was PCR amplified from 1551-2 genomic DNA using the primers tccP3flank-F (5’-CTCTCTTCTAGACGATTTTATTAAAATGCA-3’) and tccP3-R (5’-CGCGGATCCGGAATTACGCGAAGTCTTAATTCC-3’), and then cloned into XbaI and BamHI sites of pKC471, thus replacing the *tccP* gene and its upstream flanking region. Plasmids pTccP3 and pKC471 that encode Myc-tagged TccP3 and TccP proteins, respectively, were electroporated into *E. albertii* 1551-2, EHEC O157:H7 86-24 and their coisogenic mutant strains.

### Indirect Enzyme-Linked Immunosorbent Assay

Bacterial strains were grown in LB broth at 37°C overnight with shaking (200 rpm). After, bacterial cultures were inoculated (1:100) in 3 ml of DMEM and grown under the conditions described above. Culture supernatants were obtained by centrifugation at 15,700 × g for 10 min. For ELISA, the supernatants were used for coating MaxiSorp microplates (Nunc, USA). After coating (4°C for 18 h), wells were blocked with 5% non-fat milk in PBS, and incubated with anti-TccP rabbit serum (1:100) (Martins et al., 2017), followed by peroxidase-conjugated goat anti-rabbit IgG antibody (1:5,000) incubation. The assay was developed with *o*-phenylenediamine (OPD) and H_2_O_2_, and the absorbance was measured at 492 nm with a Multiskan EX ELISA reader (Labsystems, USA), using DMEM medium as a blank. Two EPEC strains, BA589 (O5:H2), which is a strong TccP2 producer, and BA1768 (O51:H40), which is a TccP and TccP2 non-producer, were used as positive and negative controls, respectively (Martins et al., 2017).

### Immunoblotting

Secreted proteins from cultures grown for 6 h in low glucose DMEM at 37°C and 5% CO_2_ were prepared as previously described (Jarvis et al., 1995). Briefly, bacterial cultures were spun down, supernatants were collected, filter sterilized using 0.22-μm filter units, and total secreted proteins were concentrated using Amicon Ultra Centrifugal filters (Millipore). Whole cell lysates were prepared by resuspending the bacterial cell pellets in lysis buffer (50 mM tris, 150 mM NaCl, 0.1%Triton-X-100, protease inhibitor cocktail, Sigma-Aldrich, pH7.4).

The proteins were separated by SDS-PAGE, transferred to polyvinylidene difluoride (PVDF) membranes, probed with rabbit polyclonal anti-EspB serum (1:5,000), mouse monoclonal anti-Myc (1:200; clone 9E40 sc-40, Santa Cruz Biotechnology) or anti-RpoA (1:10,000; Santa Cruz Biotechnology) antibodies, and visualized with enhanced chemiluminescence (Thermo Fisher) using the ChemiDoc touch imaging system (software 1.0.0.15) with Image Lab 5.2.1 software for image capture display (Bio-Rad).

### Tissue Culture Cells

Caco-2 cells were cultivated in DMEM supplemented with 10% Fetal Bovine Serum (FBS) (Gibco, USA), 1% antibiotic mixture (penicillin—10,000 U/ml and streptomycin—10 mg/ml, ThermoFisher, USA) and 1% non-essential amino acids mixture (Life Technologies, USA) in an atmosphere of 5% CO_2_ at 37°C. For quantitative assays and RNA extraction, Caco-2 cells (1 × 10^5^ cell/well) were seeded in 24 well-microplates and cultivated for 10 days. HeLa and Mouse Embryonic Fibroblast (MEF) Nck null cells (Bladt et al., 2003; Jones et al., 2006) were cultivated in DMEM supplemented with 10% FBS in the presence of 1% antibiotic mixture in an atmosphere of 5% CO_2_ at 37°C. HeLa cells transfected with Lifeact::GFP (GFP-HeLa) (Gruber and Sperandio, 2014) were grown in the presence of DMEM supplemented with 10% FBS and a mixture of 1% antibiotic mixture (penicillin—10,000 U/ml and streptomycin—10 mg/ml) + 50 µg/ml hygromycin B (Thermo Fisher, USA), and cultivated in an atmosphere of 5% CO_2_ at 37°C. For confocal fluorescent microscopic assays, GFP-HeLa cells were seeded in coverslip-bottom dishes (Corning, USA) and the same medium for 24 h. For qualitative assays, HeLa cells were seeded in 12 well-microplates, containing glass coverslips and the same medium.

### Immunofluorescence and Confocal Microscopy Assays

HeLa cells were cultured in 24-well plates with round glass coverslips or 8-well chamber slides (Lab-Tek) for approximately 48 h and then infected with bacterial strains (*E. albertii* 1551-2, O157:H7 EHEC 86-14 and their coisogenic mutants) for 6 h at 37°C in an atmosphere supplemented with 5% CO_2_. After the infections, cells were washed with PBS, fixed with 3.7% formaldehyde, permeabilized with 0.2% Triton X-100 in PBS, and blocked with PBS plus 2% BSA. For detection of TccP-Myc or TccP3-Myc, samples were then probed with mouse monoclonal anti-Myc (1:100) primary antibodies and Alexa Fluor 568-goat anti-mouse (Molecular Probes) secondary antibody at a dilution of 1:1,000. In addition, for Tir detection, rabbit polyclonal anti-Tir (1:2,000) primary antibodies and Alexa Fluor 568-goat anti-rabbit (Molecular Probes) secondary antibody at a dilution of 1:1,000 were used. Actin and DNA were stained with FITC-phalloidin (1:50) and DAPI (1:2,000), respectively. Slides were mounted with ProLong antifade and visualized with a Zeiss LSM880 confocal laser scanning microscope.

To perform the Confocal Immunofluorescence Microscopy Assay, after the adherence assay was done with HeLa cells, the preparations were fixed with 3.5% paraformaldehyde for 15 min at room temperature. Anti-TccP (1:500), and goat anti-rabbit secondary (1:250) antibodies were diluted in PGNS solution [PBS pH 7.2 with 0.1% gelatin (Sigma-Aldrich, Co., USA), 0.1% sodium azide (Sigma-Aldrich), and 0.2% saponin (Sigma-Aldrich)], and sequentially incubated at room temperature in a humidified chamber for 1 h. Slides were mounted on pH 9.0 buffered glycerol solution with 9 mM p-phenylenediamine and evaluated in a TCS SP5 II Tandem Scanner (Leica) confocal microscope with a 63× 1.40 N.A. immersion oil objective. Images were analyzed and processed with ImageJ (Schneider et al., 2012).

### Fluorescence Actin Staining Assay and F-Actin Pedestals Quantification

An overnight bacterial culture grown in LB at 37°C was inoculated (~10^7^ CFU/ml) onto HeLa or MEF Nck-/- cells that had been grown in 12-well microplates in DMEM supplemented with 2% FBS. To evaluate the kinetics of pedestal formation in cells infected with the *E. albertii* 1551-2, preparations were incubated for 2 h, 3 h, and 6 h at 37°C in an atmosphere of 5% CO_2,_ washed with PBS, and fixed with 3.7% formaldehyde for 20 min at room temperature. Coverslips were then washed with PBS and incubated with 8 µM of FITC-phalloidin (Invitrogen) solution for 30 min. After that, they were washed twice with PBS and Saline-Sodium Citrate buffer (SSC) (2x), treated with 100 µg/ml of RNAse A (Sigma-Aldrich) for 10 min, washed again with SSC (2x), incubated with 1.7 µM of propidium iodide for 10 min, and, finally, washed with SSC (2x). Images were obtained in a Zeiss Axio Vert or in a Zeiss LSM880 confocal laser scanning microscope with a 63× 1.40 N.A. immersion oil objective. Pedestal formation was quantified by randomly imaging different fields of view while recording the number of cells showing F-actin accumulation foci. Results were presented as means of percentage (%) of infected cells with F-actin or the number of pedestals per cell ± standard deviation. All samples were tested in replicates and at least two independent experiments were performed. The statistical analyses were performed with GraphPad Prism Version 8.4.2. To compare more than two means, One-way ANOVA was used, followed by the *post hoc* Tukey HSD test, in which *P* values ≤ 0.05 were considered statistically significant.

### Live Cell Imaging

Transformation of *E. albertii* 1551-2 was performed using pDP151, a recombinant plasmid that expresses mCherry (Invitrogen) and ampicillin resistance. The plasmid was introduced by electroporation in *E. albertii* competent cells, and transformants (1551-2 mCherry) were selected on LB agar with 100 μg/ml of ampicillin.

HeLa cells stably expressing Actin-GFP (Riedl et al., 2008; Gruber and Sperandio, 2014) were seeded on a coverslip-bottom dish and cultured for 24 h. *E. albertii* 1551-2 mCherry was incubated without shaking for 18 h in LB at 37°C. The cells were then infected with a 1:50 dilution of the bacterial overnight culture (10^8^ CFU/ml) in DMEM supplemented with 2% FBS and 100 μg/ml ampicillin at 37°C. After 1.5 h of incubation, the cells were washed with PBS, and then, DMEM (supplemented with 2% FBS and 100 μg/ml of ampicillin, but without Phenol Red) was added to the preparations. The cells were visualized by live-cell imaging with a Zeiss confocal microscope with a 63× 1.40 N.A. immersion oil objective. Images were taken every 2 min for 2 h.

### Kinetics of the Interaction Between *E. albertii* and Caco-2 Cells

Quantification of the 1551-2 bacteria associated with Caco-2 cells was performed as described earlier (Pacheco et al., 2014; Romão et al., 2014). Briefly, Caco-2 cells were washed with PBS, 1.0 ml of fresh medium (DMEM supplemented with 2% FBS) was added to the cell monolayers, and the cells were then inoculated with suspensions (~10^8^ CFU/ml) of bacterial cells grown overnight in LB and diluted 1:50 in DMEM, and incubated at 37°C for 1.5, 3, and 6 h. After each incubation period, cells were washed, and half of the wells were treated with gentamicin (100 µg/ml) that kills extracellular (cell surface-associated) bacteria but not intracellular bacteria (invaded bacteria, IB), while the other half of the wells remained untreated to quantify total associated bacteria (TB). After 1 h, both groups were lysed with 1% Triton X-100 and serial dilutions were plated onto MacConkey agar plates and incubated at 37°C for approximately 18 h. The resulting colonies were quantified, and invasion indexes were calculated: IB × 100/TB. All assays were performed in biological and technical triplicates, and results are presented as mean ± standard deviation.

### Quantitative PCR Assay

To study the expression of the *ler*, *escJ*, *escV*, *eae*, and *espA* genes, the 1551-2 strain was grown in LB and incubated statically at 37°C for 18 h. Bacteria (~10^8^ CFU/ml) were inoculated in Caco-2 cells in 24 well-plates for 1.5, 3, 6, and 24 h (6 h of interaction and 18 h of gentamicin treatment—100 µg/ml) at 37°C. After each incubation period, the preparation was treated with Trizol (Invitrogen, USA) and the RiboPure Bacteria Kit (Ambion) was used for total RNA extraction. cDNA synthesis was performed by the SuperScript First-Strand Synthesis kit for RT-PCR (Invitrogen, USA). The primers used to detect gene expression are listed in **Table 1**. The expression levels of each gene at different time points were compared by using the relative quantification method (Romão et al., 2014). Real-time quantification data were expressed as fold change in the expression levels of each gene at different time points. Data obtained at 3 and 6 h were compared with the 1.5 h data as a calibrator. For internal control, we used the RNA polymerase subunit alpha gene (*rpoA*). Total RNAs of all time-points were obtained from three independent assays performed in triplicate. Statistical differences were determined by the Student’s t-test, and *P* values ≤ 0.05 were considered statistically significant.

**Table 1:** Primers used in qRT-PCR assay.

Primer Identification	Sequence (5’ → 3’)	Location/Function	Reference
*ler* (F)	CGACCAGGTCTGCCCTTCT	*LEE1*	Rocha et al., 2011
*ler* (R)	GGGCGGAACTCATCGAA	*LEE1*	Rocha et al., 2011
*escJ* (F)	GGCGATGCCACTAACTGACT	*LEE2*	This study
*escJ* (R)	GCAAGCACTGTTGCTATCCA	*LEE2*	This study
*escV* (F)	GGCTCTCTTCTTCTTTATGGCTG	*LEE3*	Müller et al., 2006
*escV* (R)	CCTTTTACAAACTTCATCGCC	*LEE3*	Müller et al., 2006
*escN* (F)	GATTTCCCCCGAGTGTTTTT	*LEE3*	This study
*escN* (R)	CTGCAAGTTCTCGGGTAAGC	*LEE3*	This study
*eae* (F)	AGCAATGCCGCAGTTGATGT	*LEE5*	This study
*eae* (R)	TCCCCTTAGCTCCGGTTCCA	*LEE5*	This study
*espA* (F)	TAGTGCGAGCGCGAGTTCT	*LEE4*	This study
*espA* (R)	TCAGGCTGCGCTTCTTATGT	*LEE4*	This study
*espB* (F)	CAGAAGGCCGTTTTTGAGAG	*LEE4*	This study
*espB* (R)	CCGTTGCCTTAACCAGTGTT	*LEE 4*	This study
*espD* (F)	GCCCCTGAAGGGTTAAAGAC	*LEE4*	This study
*espD* (R)	TCTCGACGATTTTCACAACG	*LEE4*	This study
*rpoA* (F)	GCGCTCATCTTCTTCCGAAT	Housekeeping	Walters et al., 2006
*rpoA* (R)	CGCGGTCGTGGTTATGTG	Housekeeping	Walters et al., 2006

## Results

### Genetic Virulence Determinants of *E. albertii* 1551-2

**Figure 1:**
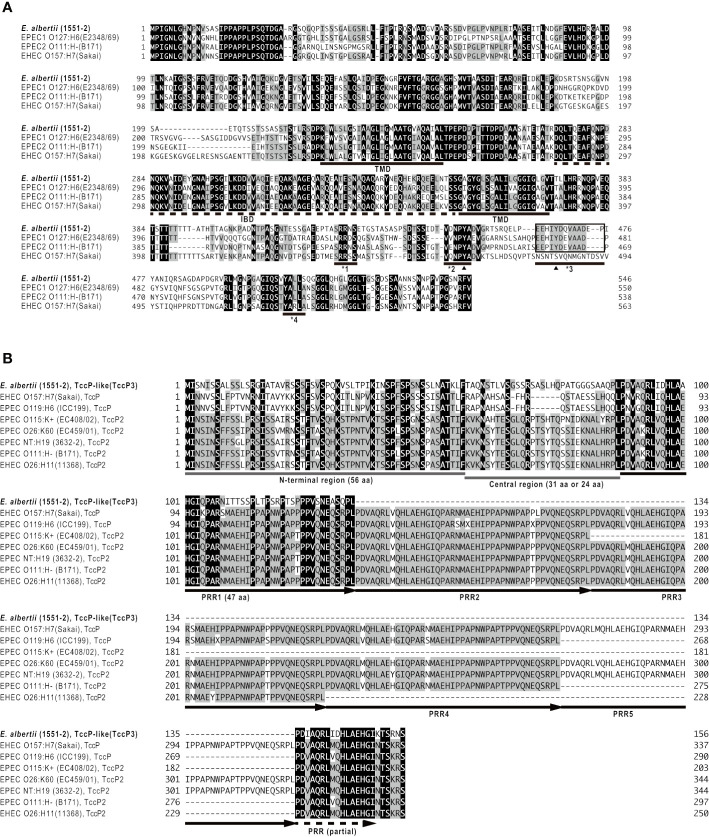
Amino acid sequence comparison of Tir **(A)** and TccP/EspF_U_ **(B)** proteins of *E. albertii* 1551-2 with known protein sequences. Strain names are shown in parentheses. In panel **(A)**, the intimin-binding domain (IBD) and two predicted transmembrane domains (TMD) of the Tir protein are indicated by dashed and solid lines, respectively. Black triangles indicate the tyrosine (Y) residues that are phosphorylated by host cell kinase(s). The underlines identified with *1, *2, *3, and *4 indicate the regions containing a sequence equivalent to EPEC Tir S_434_, O157 EHEC Tir Y_458_/EPEC Tir Y_454_, EPEC Tir Y_474_, and O157 EHEC Tir_519-524_, respectively. In panel **(B)**, the N-terminal region (56 amino acids) and central region of TccP/EspF_U_ family proteins are indicated by gray lines. Proline-rich repeats (PRRs) and a partial repeat are indicated by arrows and a dashed arrow, respectively. See **Table S1** for sequence identities of the whole proteins, N-terminal regions, central regions, and PRR1s between the proteins shown in this figure.

The LEE PAI of strain 1551-2 is integrated into the tRNA-*pheU* gene, and the core region is highly conserved when compared to those of EPEC and EHEC (data not shown), which is consistent with a previous report for multiple *E. albertii* strains (Ooka et al., 2015; Gomes et al., 2020). The Tir sequence from 1551-2 presented regions equivalent to the O157 EHEC Tir Y_458_/EPEC Tir Y_454_, as well as an equivalent to the EPEC Tir Y_474_ (**Figure 1A**), which are involved in pedestal formation *via* the Nck-independent and Nck-dependent pathways, respectively (Kenny, 1999; Campellone and Leong, 2005; Brady et al., 2007).

Twenty-one genes for non-LEE related T3SS effectors (13 families) were identified in strain 1551-2 (**Table 2**). The T3SS effector-encoding genes identified in strain 1551-2 included a *tccP*/*espF_U_* homolog (protein id: AUS68249.1). This TccP-like protein is smaller than the previously reported TccP/EspF_U_ and TccP2/EspF_M_ proteins (**Table S1**) and contained only one complete proline-rich repeat sequence (**Figure 1B**). The amino acid sequence of the whole TccP-like protein of strain 1551-2 showed 54%–55% and 50%–55% sequence identity to the TccP/EspF_U_ and TccP2/EspF_M_ proteins of EHEC and EPEC, respectively (**Table S1**). The N-terminal region and the proline-rich repeat showed 45-57% and 60-64% sequence identity to those of TccP/EspF_U_ and TccP2/EspF_M_ proteins, respectively, but the central region is totally different (**Table S1** and **Figure 1B**). These features suggest that the TccP-like protein of *E. albertii* strain 1551-2 is a novel subtype of the TccP/EspF_U_ family and was herein named as TccP3.

**Table 2:** Non-LEE T3SS effector-encoding genes identified in the *E. albertii* 1551-2 strain in comparison with prototype EHEC and EPEC strains.

Non-LEE encoded effectors	No. of genes (pseudogenes) encoding non-LEE effectors in the following genomes			
	*E. albertii* 1551-2	EHEC Sakai^a^	EPEC E2348/69^a^	EPEC B171^b^
EspI/NleA	1	1	1	1
EspJ	2	1	1	0
EspK	0	1	0	0
EspL	0	4 (1)	3 (2)	1
EspM	2	2	0	2
EspN	0	1	0	1
EspO	1	2	1 (1)	0
EspR	0	4 (1)	0	0
EspV	0	1 (1)	0	1 (1)
EspW	0	1	0	1
EspX	1	7 (1)	0	0
EspY	1	5 (1)	0	0
NleB	1	3 (1)	3 (1)	3 (2)
NleC	1	1	1	2 (2)
NleD	0	1	1	0
NleE	0	1	2	1
NleF	1	1	1	1
NleG	5	14 (5)	1	6
NleH	3	2	3 (1)	2 (1)
Cif	1	0	1 (1)	1
TccP/EspF_U_	1^c^	2 (1)	0	1
OspB	0	0	0	2 (1)
Total	21	55 (12)	19 (6)	26 (7)

a Wong et al., 2011

b Ogura et al., 2008

c A gene encoding a TccP-like protein (homologue of TccP/EspF_U_ and TccP2/EspF_M_) was identified in the 1551-2 strain (protein id: AUS68249.1).

Although ETT2 has been recognized as a cryptic second T3SS in the *E. coli*/*Shigella* lineage, Ooka et al. (2015) have shown that many *E. albertii* strains harbor an apparently intact ETT2 region and suggested that the ETT2-encoded T3SS might be involved in the virulence of this pathogen. However, in strain 1551-2, the ETT2 region has been completely deleted. This deletion was probably induced by an IS*1*-mediated transposition (**Figure S1**), but it is unknown whether this deletion occurred *in vivo* or *in vitro*.

### Kinetics of Pedestal Formation in HeLa Cells Infected With *E. albertii* 1551-2

**Figure 2:**
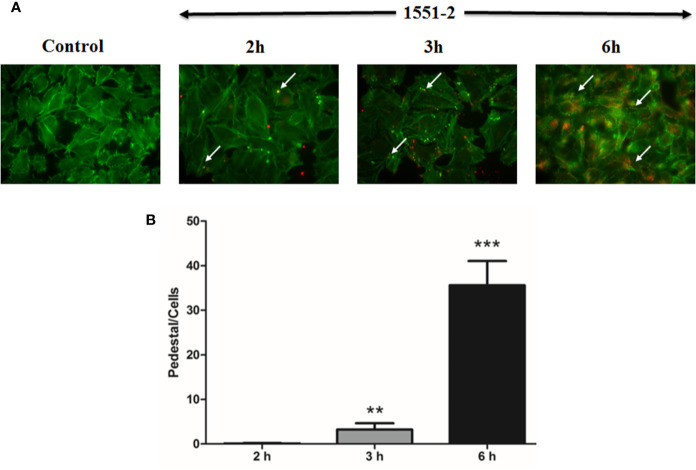
Kinetics of pedestal formation and number of pedestals produced by *E. albertii* strain 1551-2 during the initial interaction with HeLa cells. **(A)** Actin pedestals were detected after the early point (2 h) and increased in the later time-points. In green, host cell F-actin was labeled with phalloidin-FITC, and, in red, bacteria harbored the mCherry plasmid. In the Control panel, F-actin pedestals and bacteria were not detected. Arrows indicate some F-actin pedestals. **(B)** Pedestals were enumerated in ten distinct fields from the same slide and expressed as the mean number of pedestals per cell. The results at 3 and 6 h were compared with 2 h. **p < 0.01 and ***p < 0.001. Original magnification: 63 x.

The 1551-2 strain was incubated with HeLa cells, and the pedestal formed was quantified in fixed cells after 2, 3, or 6 h of interaction. Our data indicated that the bacteria rapidly induced pedestal formation at 2 h (**Figure 2A**). The number of actin pedestals increased exponentially between 3 and 6 h (**Figure 2B**). Moreover, we performed live-cell imaging of *E. albertii* 1551-2 infected HeLa cells to better probe the kinetics of actin pedestal formation. This analysis revealed that actin pedestals moved on the cell surface during all time-lapses (**Supplementary Video S1**).

### Contribution of TccP3 in the Recruitment of F-Actin in Infected HeLa Cells

**Figure 3:**
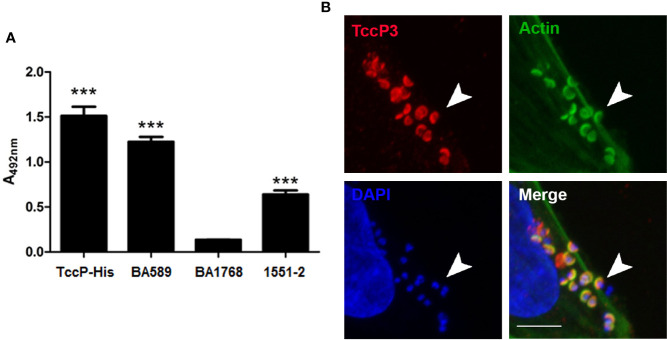
Evaluation of TccP3 production and colocalization with F-actin accumulation underneath adherent *E. albertii* 1551-2 strain. **(A)** Detection of TccP3 production by the 1551-2 strain by ELISA. The recombinant protein TccP-His and atypical EPEC BA589 (TccP2^+^) were used as positive controls, while the atypical EPEC strain BA1768 (TccP^-^/TccP2^-^) was used as a negative control. ***p < 0.001. **(B)** HeLa cells were incubated with *E. albertii* 1551-2 for 6 h, fixed and stained with anti-TccP sera, phalloidin-FITC, and DAPI, then evaluated by confocal microscopy. Actin filaments (green) clearly accumulate underneath adherent bacteria and colocalize (arrowheads) with TccP3 (red). Confocal microscopy image of a single focal plane; DAPI (blue), scale bar = 5 μm.

A novel subtype of *tccP* was identified from the whole genome sequence analysis of 1551-2 and was designated *tccP3*. We attempted to investigate if this strain would be able to produce TccP3, as well as define the role of TccP3 in triggering F-actin for pedestal formation during the infection processes. By using an anti-TccP antisera, we demonstrated that 1551-2 strain can produce TccP3 (**Figure 3A**). Moreover, we showed that the TccP3 protein co-localized with polymerized F-actin underneath adhered bacteria (**Figure 3B**). As expected, deletion of the *tccP3* gene in 1551-2 strain did not affect its ability to promote F-actin aggregation underneath adhered bacteria (data not shown), because, as previously reported, the 1551-2 strain has a functional Tir-Nck dependent pathway (Hernandes et al., 2008).

**Figure 4:**
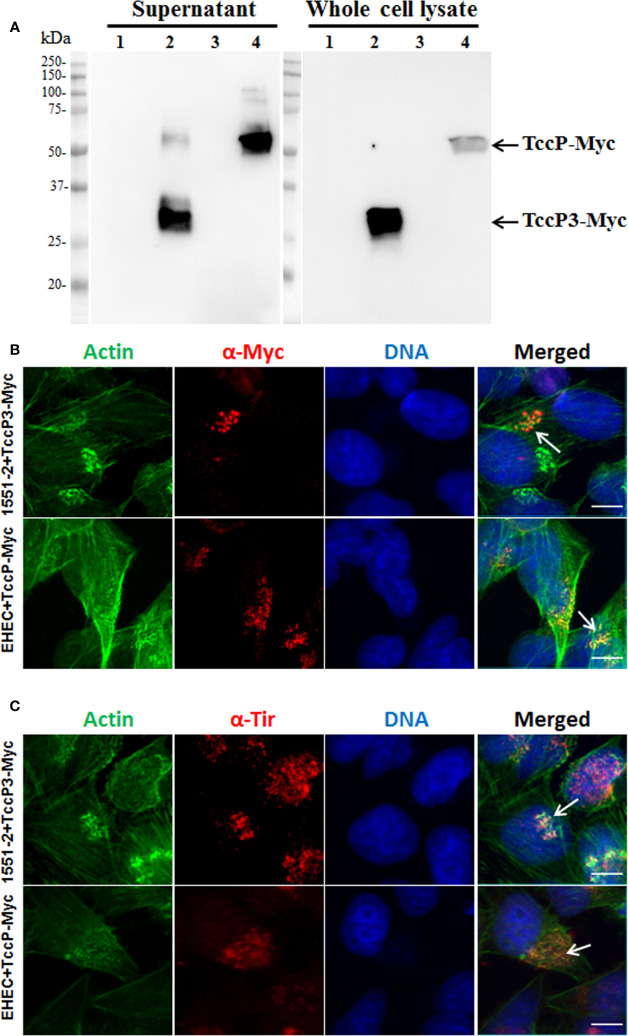
Immunoblotting and Immunofluorescence to demonstrate TccP3-Myc production and colocalization with polymerized F-actin underneath adhered bacteria in infected HeLa cells. Immunoblotting with α-Myc antibodies **(A)** with the following strains: *E. albertii* 1551-2 (lane 1), 1551-2 (pTccP3) (lane 2), EHEC 86-24 (lane 3), and EHEC (pKC471) (lane 4). Immunofluorescence with α-Myc **(B)** and α-Tir **(C)** antibodies demonstrating the colocalization (arrowheads) of TccP3-Myc and Tir proteins with polymerized F-actin in infected epithelial cells. Scale bar = 10 μm.

To investigate the contribution of TccP3 in the formation of F-actin pedestals, the *tccP3* gene was cloned in frame with a Myc tag, generating the recombinant plasmid pTccP3. We demonstrated that 1551-2 harboring this plasmid produced the TccP3-Myc fused protein (**Figure 4A**), as well as that TccP3-Myc (**Figure 4B**) and Tir (**Figure 4C**) proteins co-localized with polymerized F-actin in infected cells. Immunoblotting with anti-EspB and anti-RpoA antibodies, used as controls for the secreted protein preparation and bacterial cell lysate, respectively, are shown in **Figure S2**. Distinct to what was observed in HeLa cells infected with EHEC 86-24 (pKC471), where a perfect colocalization between F-actin and TccP-Myc can be seen, in cells infected with the 1551-2 (pTccP3) strain, one can observe the staining of F-actin colocalizing or not with TccP3-Myc, which may be explained by the fact that strain 1551-2 has a functional Tir-Nck-dependent pathway (Hernandes et al., 2008).

**Figure 5:**
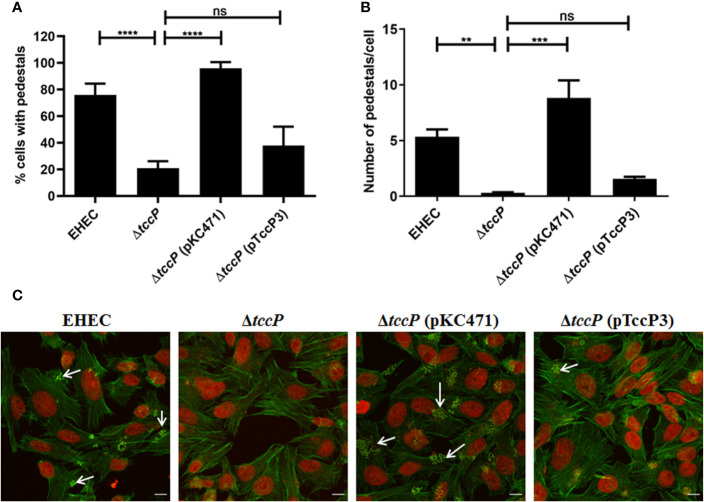
Evaluation of the efficiency in F-actin polymerization in HeLa cells due to TccP3 production in the EHECΔ*tccP* background. The efficiency of the TccP3 produced by *E. albertii* 1551-2 strain to promote F-actin aggregation was evaluated by quantitative assays to determine the percentage (%) of cell-associated bacteria with intense F-actin staining **(A)** and number of sites of F-actin staining in epithelial cells **(B)** infected with the strains: O157:H7 EHEC 86-24, EHEC 86-24Δ*tccP*, EHEC 86-24Δ*tccP* (pKC471), and EHEC 86-24Δ*tccP* (pTccP3). Representative images used in quantitative fluorescence actin staining (FAS) assays are shown in panel **(C)** and arrowheads indicate intense F-actin staining. These data demonstrate that TccP3 does not restore the efficiency of the EHEC 86-24 strain to promote F-actin accumulation underneath adherent bacteria. **p < 0.01 and ***p < 0.001, and ns: not significant. Scale bar = 10 μm.

Further, we decided to evaluate the ability of TccP3 to trigger F-actin in a Tir-Nck independent pathway by using an O157:H7 EHEC 86-24 strain. First, we demonstrated that the TccP3-Myc and Tir proteins co-localized with polymerized F-actin in infected epithelial cells (**Figure S3A**), similar to that observed with the positive control TccP-Myc (**Figure S3B**). By comparing in quantitative assays, the EHEC mutant 86-24Δ*tccP* with its complemented counterpart, 86-24Δ*tccP* (pTccP3), we observed only a very slight increase in the number of infected cells with visible F-actin staining (**Figure 5A**), as well as in the number of sites corresponding to F-actin staining per cell (**Figure 5B**), with these differences being not statistically significant. Representative images used to perform the quantitative FAS assays are given in **Figure 5C**.

Since *E. albertii* and EHEC are AE pathogens of different species, we then hypothesized that TccP3 could possess a distinct mechanism from TccP/TccP2 to trigger F-actin during the infection processes in the host cell. To test this hypothesis, we used Nck-/- MEF cells infected with the strains EHEC 86-24 and *E. albertii* 1551-2, and their respective mutants in the *tccP* and *tccP3* genes, to assess their ability to trigger F-actin underneath adherent bacteria in the absence of Nck. Surprisingly, the 1551-2Δ*tccP3* did not lose the ability to trigger F-actin, suggesting that the 1551-2 strain use an Nck-independent pathway to form F-actin pedestals, in which TccP3 does not appear to perform any significant contribution (**Figure S4**). As expected, the EHEC 86-24Δ*tccP* mutant strain did not form F-actin pedestals in the absence of Nck (**Figure S4**). Together, these data suggest that TccP3 may only weakly trigger F-actin to promote pedestal formation, or that it may not be associated with establishment of this phenotype *in vitro*.

### *E. albertii* 1551-2 Invasion in Caco-2 Cells Depends on Efficient Adherence During the Initial Stages of Bacteria-Cell Interaction

**Figure 6:**
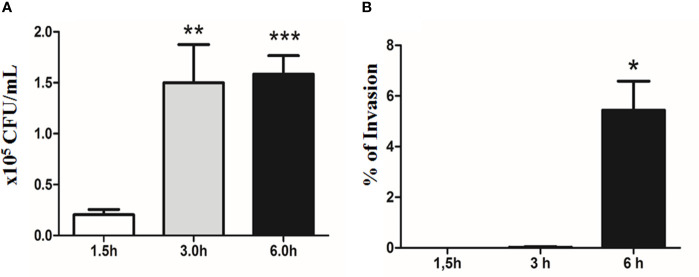
Quantitative bacterial adherence and invasion indexes of *E. albertii* 1551-2 in differentiated Caco-2 cells at 1.5, 3, and 6 h. **(A)** *E. albertii* 1551-2 can associate with Caco-2 cells efficiently within 3 h after infection. The number of associated bacteria did not change between 3 and 6 h, suggesting that, at 3 h, bacteria have occupied most of the host sites available *in vitro* under this condition. **p < 0.01 and ***p < 0.001. **(B)** No intracellular bacteria were recovered after 1.5 h of infection. A low number of bacteria was detected at 3 h (0.2% of invasion). *p < 0.05. The results at 3 and 6 h were compared with those obtained in 1.5 h.

*E. albertii* 1551-2 progressively adhered to Caco-2 cell surfaces during the first 3 h of the assay (2.04 ± 1.38 × 10^4^ and 1.50 ± 0.83 × 10^5^ CFU/ml in 1.5 and 3 h, respectively). However, the CFUs did not change substantially between 3 h and 6 h (1.58 ± 0.77 × 10^5^ CFU/ml) (**Figure 6A**).

The invasion started around 3 h of contact, with only a few internalized bacteria being recovered at this time-point, reflecting a very low index of invasion (0.02 ± 0.04%). However, by 6 h, the invasion index significantly increased to 5.44 ± 1.14% (**Figure 6B**).

### Expression Levels of the LEE Genes Are Constant During the Initial Stages of Bacterial Interaction With Differentiated Intestinal Caco-2 Cells

**Figure 7:**
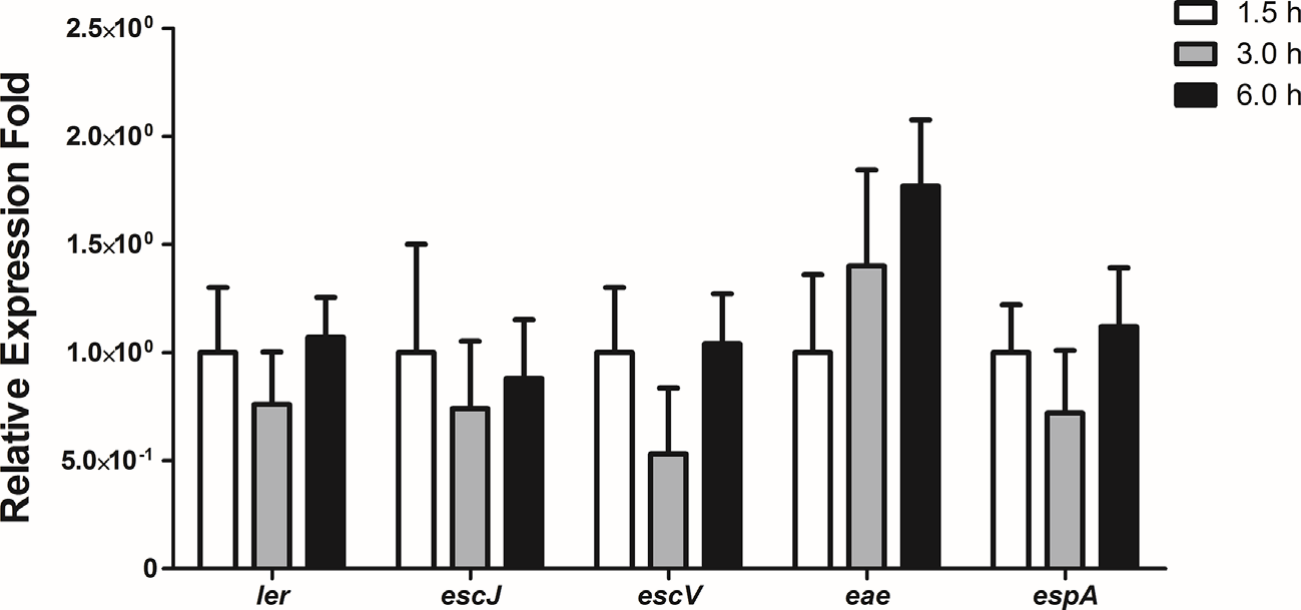
Relative gene expression of the LEE region operons during the interaction of *E. albertii* 1551-2 strain with differentiated intestinal Caco-2 cells at the time-points 1.5, 3, and 6 h. No differences were detected in the expression of all genes tested among the three time-points. *p* > 0.05.

To evaluate the expression levels of the LEE genes during the initial stages of the interaction of 1551-2 strain with differentiated Caco-2 cells, we performed a kinetic study of the expression of five LEE genes, each representing one of the five polycistronic LEE operons, using the following time-points: 1.5, 3, and 6 h. No statistically significant change in the expression of the LEE genes was detected during this time-course (**Figure 7**), suggesting that the T3SS and its constant expression may be necessary for efficient colonization, and subsequent events throughout the time bacteria were in contact with intestinal cells.

### The LEE Genes Are Overexpressed During Persistence Inside Caco-2 Cells

**Figure 8:**
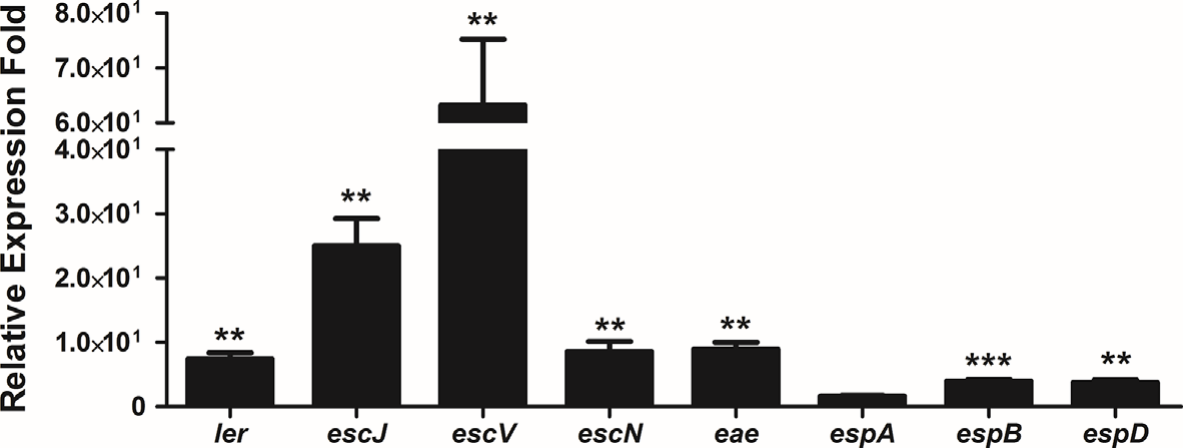
Relative gene expression of the LEE operons by internalized *E. albertii* 1551-2 strain in Caco-2 cells in the period of 24 h, as compared with internalized bacteria at 6 h. The *ler* (*LEE1*), *escJ* (*LEE2*), *escV* (*LEE3*), *escN* (*LEE3*), *eae* (*LEE5*), *espB* (*LEE4*), and *espD* (*LEE4*) genes were overexpressed by intracellular bacteria at least 4-fold at 24 h in comparison with 6 h. No alteration of *espA* (*LEE4*) expression was detected. **p < 0.01 and ***p < 0.001.

Pacheco and co-workers (2014) reported that strain 1551-2 was able to persist and multiply in the intracellular environment up to 24 h when the population was twice as high when compared to the 6 h time point, and the Caco-2 monolayers were preserved during that period. In addition, transmission electron microscopy images showed that intracellular bacteria were imaged on top of actin pedestal-like structures inside vacuoles (Pacheco et al., 2014). Therefore, we decided to evaluate the expression of the LEE genes after 24 h of infection by qRT-PCR. Except for *espA*, the expression of the eight LEE genes investigated significantly increased at 24 h in comparison with 6 h (**Figure 8**). This suggests that the LEE-encoded T3SS may be necessary for 1551-2 strain to persist for more extended periods inside the host cell.

## Discussion

In this study, we reported genomic features of the *E. albertii* strain 1551-2, which was formerly classified as atypical EPEC (Vieira et al., 2001; Hernandes et al., 2006), and evaluated the expression of the LEE along the process of colonization of differentiated intestinal Caco-2 cells.

By studying the dynamics of the interaction of strain 1551-2 with epithelial cells, in this study, we found actin pedestals moving on the HeLa cell surface during all time-lapses studied. Similar behavior was reported recently for an EHEC strain and a typical EPEC strain (Velle and Campellone, 2017). In addition, it was shown that EHEC extracellular motility and cell-to-cell transmission are driven by TccP/EspF_U_-mediated actin assembly (Velle and Campellone, 2017). These observations suggest that actin pedestal mobility may be necessary for bacterial internalization, and perhaps for bacterial persistence within cells.

Extending the genomic analyses of the 1551-2 strain, we have identified a gene encoding a new variant of the TccP/EspF_U_ family, which was termed *tccP3*. Although we have accumulated evidence that the TccP3 protein is produced by 1551-2 strain, and that this non-LEE effector is translocated to infected host cells and co-localizes with both polymerized F-actin and Tir, apparently TccP3 was not sufficient to efficiently trigger F-actin to form pedestals *via* a Tir-Nck independent pathway in the EHECΔ*tccP* background. A hypothesis that can explain the previously mentioned data is the fact that the proline-rich segment ^27^IPPAPNWPAPTPP^39^, which harbors the motifs responsible for linking the TccP/EspF_U_ bacterial protein to the SH3 domain of the host protein IRTKS (Aitio et al., 2012), is only partially conserved in the 1551-2 strain. Additionally, in the same study, the authors have identified that the amino acid tryptophan (W), present in the proline-rich segment ^27^IPPAPN**W**PAPTPP^39^ of the TccP/EspF_U_ protein, is critical for its high affinity to the IRTKS/IRSp53 host linker (Aitio et al., 2012). The 1551-2 strain lacks this tryptophan residue, harboring an arginine (R) in the corresponding position. All these observations allowed us to hypothesize that TccP3 probably has a low affinity to IRTKS/IRSp53, and thus is not efficient enough to trigger F-actin polymerization underneath adhered bacteria to form pedestals in the infected cells. It is also important to consider as a hypothesis the fact that an *in trans* complementation assays of the EDL933Δ*tccP* mutant strain with a plasmid encoding the TccP protein harboring only the N-terminus translocation domain and one proline-rich repeat, similar to TccP3, was unable to complement the loss of actin polymerization activity, distinct to that observed with the TccP protein with two proline-rich repeats (Garmendia et al., 2006).

The assays carried out with Nck-null MEF cells, infected with wildtype *E. albertii* 1551-2 and its *tccP3* derivative mutant, reinforced the data obtained with the *in trans* complementation of EHEC 86-24Δ*tccP* with *tccP3*, thus excluding a potential contribution of the TccP3 effector to polymerize F-actin underneath adherent bacteria. Of importance and surprisingly, 1551-2Δ*tccP3* triggered F-actin polymerization in an Nck-independent pathway, indicating that 1551-2 strain can use both Nck-dependent, as previously observed (Hernandes et al., 2008), and Nck-independent pathways to form F-actin pedestals in infected host cells. However, the bacterial effector(s) involved in the Nck-independent F-actin recruitment remains unclear and will certainly be addressed in future studies. Very recently, it has been demonstrated that TccP/EspF_U_ can reverse the anti-inflammatory response induced by EPEC in epithelial cells (Martins et al., 2020). However, if TccP3 could act similarly to modulate the inflammatory response of the host cell, it is a question that remains to be explored in further studies. Regarding TccP3, it may be noteworthy that our preliminary analysis of the prevalence of *tccP3* in 243 *E. albertii* genomes (Ooka et al., 2019) identified only five *tccP3*-positive strains (including strain 1551-2). However, as there is considerable difficulty in searching for *tccP* family members in draft genome sequences due to the presence of internal repeats, more detailed analyses are required to determine the precise prevalence of *tccp3* genes and their intactness.

Even though we have previously demonstrated the ability of 1551-2 strain to invade and persist in differentiated intestinal Caco-2 cells (Pacheco et al., 2014; Yamamoto et al., 2017), and the critical contribution of the interaction between the LEE-encoded intimin-Tir proteins for the establishment of this invasive phenotype (Hernandes et al., 2008; Hernandes et al., 2013; Yamamoto et al., 2017), a kinetic study of these events and the LEE expression levels during the different stages of the infection processes were hitherto unknown. Quantitative assays of the interaction of 1551-2 strain with Caco-2 cells showed important key points to assist us in understanding these processes. A good example of this was the definition of two essential time points to study the processes of adhesion and invasion of host cells by this pathogen (3 and 6 h, respectively). Corroborating our previous observation that interaction between the LEE encoded intimin and Tir proteins is an important step in the adhesion process (Hernandes et al., 2013) and essential for invasion (Hernandes et al., 2008; Yamamoto et al., 2017) of host cells infected with the 1551-2 strain, we observed in this study that all five operons of the LEE region were consistently expressed during these stages of the infectious process.

Importantly, LEE genes are up-regulated during the 1551-2 strain intracellular stage, pointing to the importance of their expression in maintenance of the strain inside the host epithelial cells. These data should be considered together with findings from our previous study with the 1551-2 strain. First, we reported that this strain was located within individual vacuoles surrounded by polymerized F-actin inside infected HeLa cells (Hernandes et al., 2008). Further, transmission electron microscopy of infected Caco-2 cells also showed F-actin rich pedestals underneath adherent bacteria associated with the cytoplasmic vacuole membrane. *Salmonella*-containing vacuoles are also surrounded by polymerized F-actin, with this morphological aspect of vacuoles being induced by proteins encoded by genes located in the SPI-2 (*Salmonella* pathogenicity island 2) (Guiney and Lesnick, 2005). Vacuole-containing bacteria surrounded by polymerized F-actin were also observed in the intracellular compartment of Caco-2 cells infected by atypical EPEC E110019 (Bulgin et al., 2009), obtained from one of the most severe diarrheal outbreaks due to diarrheagenic *E. coli* infection in 650 individuals in a school and 137 associated household members with diarrheal disease in Finland (Viljanen et al., 1990). The biological role of this event may be related to allowing the maintenance and replication of this pathogen within the cellular environment, thus protecting these bacteria from the host’s defenses.

In summary, we showed the constant expression of the LEE genes through the distinct time-points of infection, thus reinforcing the importance of the LEE-encoded T3SS proteins during adhesion, invasion and bacterial persistence of *E. albertii* 1551-2 strain in the host cells. A better understanding of the roles of *E. albertii* T3SS may help to elucidate the versatility of this system for AE pathogens since AE bacteria are usually extracellular and rarely reach the intracellular environment.

## Data Availability Statement

The raw data supporting the conclusions of this article will be made available by the authors, without undue reservation, to any qualified researcher.

## Author Contributions

TG, RH, TH, and VS conceptualized the study. FR, FM, TO, FS, DY, AB-M, NJ, and WE contributed to the formal analysis. TG, TH, and VS were responsible for the funding acquisition. FR, FM, RH, TO, FS, DY, and AB-M carried out the investigation. TG, TH, RH, and VS helped with the project administration. TG, TH, WE, RH, and VS supervised the study. TG, TH, TO, WE, RH, and VS validated the study. FR, RH, and TG wrote the original draft. FR, FM, RH, TO, FS, DY, AB-M, TH, WE, NJ, VS, and TG reviewed and edited the manuscript. All authors contributed to the article and approved the submitted version.

## Funding

This work was supported by Coordenação de Aperfeiçoamento de Pessoal de Nível Superior (CAPES) (grant 99999.009868/2014-03), Fundação de Amparo à Pesquisa do Estado de São Paulo (FAPESP) (grant 2011/12664-5), National Council for Scientific and Technological Development (CNPq) (grant 141586/2013-3), National Institutes of Health (NIH) (grant AI053067), JSPS KAKENHI (grant 16K08781) and Grants-in-Aid for Scientific Research from the Research Program on Emerging and Re-emerging Infectious Diseases from the Japan Agency for Medical Research and Development (AMED).

## Conflict of Interest

The authors declare that the research was conducted in the absence of any commercial or financial relationships that could be construed as a potential conflict of interest.

## Supplementary Material

Supplementary Table 1 Amino acid sequence identity among the TccP, TccP2, and TccP3 proteins of 1551-2 *E. albertii* and *E. coli* strains.

Supplementary Figure 1 Structural comparison of the ETT2 region of *E. albertii* CB9786 strain and the corresponding region of *E. albertii* 1551-2 strain. ETT2 was not detected in the 1551-2 strain.

Supplementary Figure 2 Immunoblotting with anti-EspB **(A)** and anti-RpoA **(B)** antibodies, used as controls for the secreted protein preparation and bacterial cell lysate, respectively. Strains tested: *E. albertii* 1551-2 (lane 1), 1551-2 (pTccP3) (lane 2), EHEC 86-24 (lane 3), and EHEC (pKC471) (lane 4). The T3SS-dependent effector EspB and RpoA, the α subunit of the RNA polymerase core enzyme, were detected in all strains tested.

Supplementary Figure 3 Immunofluorescence showing the colocalization of polymerized F-actin with Tir and TccP3-Myc proteins. Note the colocalization (arrowheads) of polymerized F-actin with Tir and TccP3/TccP-Myc proteins in HeLa cells infected with EHEC 86-24Δ*tccP* strain harboring pTccP3 **(A)** or pKC471 **(B)** plasmids, respectively. Scale bar = 10 μm.

Supplementary Figure 4 Evaluation of the participation of TccP3 in F-actin polymerization in Nck-/- Mouse embryonic fibroblast (MEF) cells. The following strains were tested: *E. albertii* 1551-2 **(A)**, 1551-2Δ*tccP3* **(B)**, EHEC 86-24 **(C)**, and EHECΔ*tccP* **(D)**. Lack of TccP3 production did not affect the ability of the 1551-2Δ*tccP3* mutant strain in triggering F-actin polymerization underneath adherent bacteria in an Nck-independent manner. Arrows indicate colocalization of adherent bacteria with polymerized F-actin. Scale bar = 10 μm.

Supplementary Video Sheet 1 Live cell imaging of the interaction between *E. albertii* 1551-2 and HeLa cells. After 1.5 h of interaction between *E. albertii* and HeLa cells, the images were acquired every 2 min for 2 h. The actin pedestals increased in size and moved around on the surfaces of the cells during the observed time.

## References

[B1] Aitio O. Hellman M. Skehan B. Kesti T. Leong J. M. Saksela K. (2012). Enterohaemorrhagic *Escherichia coli* exploits a tryptophan switch to hijack host f-actin assembly. Structure 20, 1692–1703. 10.1016/j.str.2012.07.015 22921828 PMC3472031

[B2] Berdichevsky T. Friedberg D. Nadler C. Rokney A. Oppenheim A. Rosenshine I. (2005). Ler is a negative autoregulator of the LEE1 operon in enteropathogenic *Escherichia coli* . J. Bacteriol. 187, 349–357. 10.1128/JB.187.1.349-357.2005 15601719 PMC538822

[B3] Bladt F. Aippersbach E. Gelkop S. Strasser G. A. Nash P. Tafuri A. (2003). The murine Nck SH2/SH3 adaptors are important for the development of mesoderm-derived embryonic structures and for regulating the cellular actin network. Mol. Cell. Biol. 23, 4586–4597. 10.1128/mcb.23.13.4586-4597.2003 12808099 PMC164855

[B4] Brady M. J. Campellone K. G. Ghildiyal M. Leong J. M. (2007). Enterohaemorrhagic and enteropathogenic *Escherichia coli* Tir proteins trigger a common Nck-independent actin assembly pathway. Cell. Microbiol. 9, 2242–2253. 10.1111/j.1462-5822.2007.00954.x 17521329

[B5] Bulgin R. Arbeloa A. Goulding D. Dougan G. Crepin V. F. Raymond B. (2009). The T3SS effector EspT defines a new category of invasive enteropathogenic *E. coli* (EPEC) which form intracellular actin pedestals. PloS Pathog. 5, e1000683. 10.1371/journal.ppat.1000683 20011125 PMC2782363

[B6] Bustamante V. H. Santana F. J. Calva E. Puente J. L. (2001). Transcriptional regulation of type III secretion genes in enteropathogenic *Escherichia coli*: Ler antagonizes H-NS-dependent repression. Mol. Microbiol. 39, 664–678. 10.1046/j.1365-2958.2001.02209.x 11169107

[B7] Campellone K. G. Robbins D. Leong J. M. (2004). EspFu is a translocated EHEC effector that interacts with Tir and N-WASP and promotes Nck-independent actin assembly. Dev. Cell 7, 217–228. 10.1016/j.devcel.2004.07.004 15296718

[B8] Campellone K. G. Leong J. M. (2005). Nck-independent actin assembly is mediated by two phosphorylated tyrosines within enteropathogenic *Escherichia coli* Tir. Mol. Microbiol. 56, 416–432. 10.1111/j.1365-2958.2005.04558.x 15813734

[B9] Dean P. Kenny B. (2009). The effector repertoire of enteropathogenic *E. coli*: ganging up on the host cell. Curr. Opin. Microbiol. 12, 101–109. 10.1016/j.mib.2008.11.006 19144561 PMC2697328

[B10] Deng W. Puente J. L. Gruenheid S. Li Y. Vallance B. A. Vazquez A. (2004). Dissecting virulence: systematic and functional analyses of a pathogenicity island. Proc. Natl. Acad. Sci. U.S.A. 101, 3597–3602. 10.1073/pnas.0400326101 14988506 PMC373508

[B11] Elliott S. J. Wainwright L. A. Mcdaniel T. K. Jarvis K. G. Deng Y. K. Lai L. C. (1998). The complete sequence of the locus of enterocyte effacement (LEE) from enteropathogenic *Escherichia coli* E2348/69. Mol. Microbiol. 28, 1–4. 10.1046/j.1365-2958.1998.00783.x 9593291

[B12] Garmendia J. Phillips A. D. Carlier M. F. Chong Y. Schuller S. Marches O. (2004). TccP is an enterohaemorrhagic *Escherichia coli* O157:H7 type III effector protein that couples Tir to the actin-cytoskeleton. Cell. Microbiol. 6, 1167–1183. 10.1111/j.1462-5822.2004.00459.x 15527496

[B13] Garmendia J. Carlier M. F. Egile C. Didry D. Frankel G. (2006). Characterization of TccP-mediated N-WASP activation during enterohaemorrhagic *Escherichia coli* infection. Cell. Microbiol. 8, 1444–1455. 10.1111/j.1462-5822.2006.00723.x 16922863

[B14] Gaytan M. O. Martinez-Santos V. II Soto E. Gonzalez-Pedrajo B. (2016). Type Three Secretion System in Attaching and Effacing Pathogens. Front. Cell. Infect. Microbiol. 6:129:129. 10.3389/fcimb.2016.00129 27818950 PMC5073101

[B15] Gomes T. A. T. Ooka T. Hernandes R. T. Yamamoto D. Hayashi T. (2020). *Escherichia albertii* Pathogenesis. EcoSal Plus Press. 9, 1–18. 10.1128/ecosalplus.ESP-0015-2019 PMC11168576 32588811

[B16] Gruber C. C. Sperandio V. (2014). Posttranscriptional control of microbe-induced rearrangement of host cell actin. mBio 5, e01025–e01013. 10.1128/mBio.01025-13 24425733 PMC3903284

[B17] Guiney D. G. Lesnick M. (2005). Targeting of the actin cytoskeleton during infection by *Salmonella* strains. Clin. Immunol. 114, 248–255. 10.1016/j.clim.2004.07.014 15721835

[B18] Hayashi T. Makino K. Ohnishi M. Kurokawa K. Ishii K. Yokoyama K. (2001). Complete genome sequence of enterohemorrhagic *Escherichia coli* O157:H7 and genomic comparison with a laboratory strain K-12. DNA Res. 8, 11–22. 10.1093/dnares/8.1.11 11258796

[B19] Hernandes R. T. Vieira M. A. M. Carneiro S. M. Salvador F. A. Gomes T. A. T. (2006). Characterization of atypical enteropathogenic *Escherichia coli* strains that express typical localized adherence in HeLa cells in the absence of the bundle-forming pilus. J. Clin. Microbiol. 44, 4214–4217. 10.1128/JCM.01022-06 16957035 PMC1698306

[B20] Hernandes R. T. Silva R. M. Carneiro S. M. Salvador F. A. Fernandes M. C. Padovan A. C. (2008). The localized adherence pattern of an atypical enteropathogenic *Escherichia coli* is mediated by intimin omicron and unexpectedly promotes HeLa cell invasion. Cell. Microbiol. 10, 415–425. 10.1111/j.1462-5822.2007.01054.x 17910741

[B21] Hernandes R. T. De La Cruz M. A. Yamamoto D. Giron J. A. Gomes T. A. T. (2013). Dissection of the role of pili and type 2 and 3 secretion systems in adherence and biofilm formation of an atypical enteropathogenic *Escherichia coli* strain. Infect. Immun. 81, 3793–3802. 10.1128/IAI.00620-13 23897608 PMC3811761

[B22] Huys G. Cnockaert M. Janda J. M. Swings J. (2003). *Escherichia albertii* sp. nov., a diarrhoeagenic species isolated from stool specimens of Bangladeshi children. Int. J. Syst. Evol. Microbiol. 53, 807–810. 10.1099/ijs.0.02475-0 12807204

[B23] Hyma K. E. Lacher D. W. Nelson A. M. Bumbaugh A. C. Janda J. M. Strockbine N. A. (2005). Evolutionary genetics of a new pathogenic *Escherichia* species: *Escherichia albertii* and related *Shigella boydii* strains. J. Bacteriol. 187, 619–628. 10.1128/JB.187.2.619-628.2005 15629933 PMC543563

[B24] Ide T. Laarmann S. Greune L. Schillers H. Oberleithner H. Schmidt M. A. (2001). Characterization of translocation pores inserted into plasma membranes by type III-secreted Esp proteins of enteropathogenic *Escherichia coli* . Cell. Microbiol. 3, 669–679. 10.1046/j.1462-5822.2001.00146.x 11580752

[B25] Iguchi A. Thomson N. R. Ogura Y. Saunders D. Ooka T. Henderson I. R. (2009). Complete genome sequence and comparative genome analysis of enteropathogenic *Escherichia coli* O127:H6 strain E2348/69. J. Bacteriol. 191, 347–354. 10.1128/JB.01238-08 18952797 PMC2612414

[B26] Jarvis K. G. Giron J. A. Jerse A. E. Mcdaniel T. K. Donnenberg M. S. Kaper J. B. (1995). Enteropathogenic *Escherichia coli* contains a putative type III secretion system necessary for the export of proteins involved in attaching and effacing lesion formation. Proc. Natl. Acad. Sci. U.S.A. 92, 7996–8000. 10.1073/pnas.92.17.7996 7644527 PMC41273

[B27] Jerse A. E. Yu J. Tall B. D. Kaper J. B. (1990). A genetic locus of enteropathogenic *Escherichia coli* necessary for the production of attaching and effacing lesions on tissue culture cells. Proc. Natl. Acad. Sci. U.S.A. 87, 7839–7843. 10.1073/pnas.87.20.7839 2172966 PMC54845

[B28] Jones N. Blasutig I. M. Eremina V. Ruston J. M. Bladt F. Li H. (2006). Nck adaptor proteins link nephrin to the actin cytoskeleton of kidney podocytes. Nature 440, 818–823. 10.1038/nature04662 16525419

[B29] Kenny B. Devinney R. Stein M. Reinscheid D. J. Frey E. A. Finlay B. B. (1997). Enteropathogenic *E. coli* (EPEC) transfers its receptor for intimate adherence into mammalian cells. Cell 91, 511–520. 10.1016/s0092-8674(00)80437-7 9390560

[B30] Kenny B. (1999). Phosphorylation of tyrosine 474 of the enteropathogenic *Escherichia coli* (EPEC) Tir receptor molecule is essential for actin nucleating activity and is preceded by additional host modifications. Mol. Microbiol. 31, 1229–1241. 10.1046/j.1365-2958.1999.01265.x 10096089

[B31] Knutton S. Baldwin T. Williams P. H. Mcneish A. S. (1989). Actin accumulation at sites of bacterial adhesion to tissue culture cells: basis of a new diagnostic test for enteropathogenic and enterohemorrhagic *Escherichia coli* . Infect. Immun. 57, 1290–1298. 10.1128/IAI.57.4.1290-1298.1989 2647635 PMC313264

[B32] Knutton S. Rosenshine I. Pallen M. J. Nisan I. Neves B. C. Bain C. (1998). A novel EspA-associated surface organelle of enteropathogenic *Escherichia coli* involved in protein translocation into epithelial cells. EMBO J. 17, 2166–2176. 10.1093/emboj/17.8.2166 9545230 PMC1170561

[B33] Kotloff K. L. Nataro J. P. Blackwelder W. C. Nasrin D. Farag T. H. Panchalingam S. (2013). Burden and aetiology of diarrhoeal disease in infants and young children in developing countries (the Global Enteric Multicenter Study, GEMS): a prospective, case-control study. Lancet 382, 209–222. 10.1016/S0140-6736(13)60844-2 23680352

[B34] Martins F. H. Nepomuceno R. Piazza R. M. F. Elias W. P. (2017). Phylogenetic distribution of tir-cytoskeleton coupling protein (*tccP* and *tccP2*) genes in atypical enteropathogenic *Escherichia coli* . FEMS Microbiol. Lett. 364, 1–7. 10.1093/femsle/fnx101 28505295

[B35] Martins F. H. Kumar A. Abe C. M. Carvalho E. Nishiyama-Jr M. Xing C. (2020). EspFu-Mediated Actin Assembly Enhances Enteropathogenic *Escherichia coli* Adherence and Activates Host Cell Inflammatory Signaling Pathways. mBio 11, e00617–e00620. 10.1128/mBio.00617-20 32291304 PMC7157822

[B36] McDaniel T. K. Jarvis K. G. Donnenberg M. S. Kaper J. B. (1995). A genetic locus of enterocyte effacement conserved among diverse enterobacterial pathogens. Proc. Natl. Acad. Sci. U.S.A. 92, 1664–1668. 10.1073/pnas.92.5.1664 7878036 PMC42580

[B37] Mellies J. L. Elliott S. J. Sperandio V. Donnenberg M. S. Kaper J. B. (1999). The Per regulon of enteropathogenic *Escherichia coli*: identification of a regulatory cascade and a novel transcriptional activator, the locus of enterocyte effacement (LEE)-encoded regulator (Ler). Mol. Microbiol. 33, 296–306. 10.1046/j.1365-2958.1999.01473.x 10411746

[B38] Moon H. W. Whipp S. C. Argenzio R. A. Levine M. M. Giannella R. A. (1983). Attaching and effacing activities of rabbit and human enteropathogenic *Escherichia coli* in pig and rabbit intestines. Infect. Immun. 41, 1340–1351. 10.1128/IAI.41.3.1340-1351.1983 6350186 PMC264644

[B39] Müller D. Hagedorn P. Brast S. Heusipp G. Bielaszewska M. Friedrich A. W. (2006). Rapid identification and differentiation of clinical isolates of enteropathogenic *Escherichia coli* (EPEC), atypical EPEC, and Shiga toxin-producing *Escherichia coli* by a one-step multiplex PCR method. J. Clin. Microbiol. 44, 2626–2629. 10.1128/JCM.00895-06 16825399 PMC1489516

[B40] Oaks J. L. Besser T. E. Walk S. T. Gordon D. M. Beckmen K. B. Burek K. A. (2010). *Escherichia albertii* in wild and domestic birds. Emerg. Infect. Dis. 16, 638–646. 10.3201/eid1604.090695 20350378 PMC3321939

[B41] Ogura Y. Abe H. Katsura K. Kurokawa K. Asadulghani M. Iguchi A. (2008). Systematic identification and sequence analysis of the genomic islands of the enteropathogenic *Escherichia coli* strain B171-8 by the combined use of whole-genome PCR scanning and fosmid mapping. J. Bacteriol. 190, 6948–6960. 10.1128/JB.00625-08 18757547 PMC2580699

[B42] Ooka T. Ogura Y. Katsura K. Seto K. Kobayashi H. Kawano K. (2015). Defining the Genome Features of *Escherichia albertii*, an Emerging Enteropathogen Closely Related to *Escherichia coli* . Genome Biol. Evol. 7, 3170–3179. 10.1093/gbe/evv211 26537224 PMC4700944

[B43] Ooka T. Seto K. Ogura Y. Nakamura K. Iguchi A. Gotoh Y. (2019). O-antigen biosynthesis gene clusters of *Escherichia albertii*: their diversity and similarity to *Escherichia coli* gene clusters and the development of an O-genotyping method. Microb Genom 5, e000314. 10.1099/mgen.0.000314 PMC6927306 31738701

[B44] Pacheco V. C. Yamamoto D. Abe C. M. Hernandes R. T. Mora A. Blanco J. (2014). Invasion of differentiated intestinal Caco-2 cells is a sporadic property among atypical enteropathogenic *Escherichia coli* strains carrying common intimin subtypes. Pathog. Dis. 70, 167–175. 10.1111/2049-632X.12112 24339197

[B45] Pearson J. S. Giogha C. Lung T. W. F. Hartland E. L. (2016). The Genetics of Enteropathogenic *Escherichia coli* Virulence. Annu. Rev. Genet. 50, 493–513. 10.1146/annurev-genet-120215-035138 27893961

[B46] Riedl J. Crevenna A. H. Kessenbrock K. Yu J. H. Neukirchen D. Bista M. (2008). Lifeact: a versatile marker to visualize F-actin. Nat. Methods 5, 605–607. 10.1038/nmeth.1220 18536722 PMC2814344

[B47] Rocha S. P. Abe C. M. Sperandio V. Bando S. Y. Elias W. P. (2011). Atypical enteropathogenic *Escherichia coli* that contains functional locus of enterocyte effacement genes can be attaching-and-effacing negative in cultured epithelial cells. Infect. Immun. 79, 1833–1841. 10.1128/IAI.00693-10 21343354 PMC3088124

[B48] Romão F. T. Hernandes R. T. Yamamoto D. Osugui L. Popi A. F. Gomes T. A. T. (2014). Influence of environmental factors in the adherence of an atypical enteropathogenic *Escherichia coli* strain to epithelial cells. BMC Microbiol. 14:299. 10.1186/s12866-014-0299-y 25527183 PMC4290818

[B49] Romão F. T. Hernandes R. T. Ooka T. Hayashi T. Sperandio V. Gomes T. A. T. (2018). Complete Genome Sequence of *Escherichia albertii* Strain 1551-2, a Potential Extracellular and Intracellular Pathogen. Genome Announc. 6, e00075–18. 10.1128/genomeA.00075-18 PMC5834326 29496827

[B50] Sampaio S. C. Gomes T. A. T. Pichon C. Du Merle L. Guadagnini S. Abe C. M. (2009). The flagella of an atypical enteropathogenic *Escherichia coli* strain are required for efficient interaction with and stimulation of interleukin-8 production by enterocytes *in vitro* . Infect. Immun. 77, 4406–4413. 10.1128/IAI.00177-09 19620340 PMC2747955

[B51] Schneider C. A. Rasband W. S. Eliceiri K. W. (2012). NIH Image to ImageJ: 25 years of image analysis. Nat. Methods 9, 671–675. 10.1038/nmeth.2089 22930834 PMC5554542

[B52] Serapio-Palacios A. Finlay B. B. (2020). Dynamics of expression, secretion and translocation of type III effectors during enteropathogenic *Escherichia coli* infection. Curr. Opin. Microbiol. 54, 67–76. 10.1016/j.mib.2019.12.001 32058947

[B53] Tobe T. Beatson S. A. Taniguchi H. Abe H. Bailey C. M. Fivian A. (2006). An extensive repertoire of type III secretion effectors in *Escherichia coli* O157 and the role of lambdoid phages in their dissemination. Proc. Natl. Acad. Sci. U.S.A. 103, 14941–14946. 10.1073/pnas.0604891103 16990433 PMC1595455

[B54] Velle K. B. Campellone K. G. (2017). Extracellular motility and cell-to-cell transmission of enterohemorrhagic *E. coli* is driven by EspFu-mediated actin assembly. PloS Pathog. 13, e1006501. 10.1371/journal.ppat.1006501 28771584 PMC5557606

[B55] Vieira M. A. M. Andrade J. R. Trabulsi L. R. Rosa A. C. Dias A. M. Ramos S. R. (2001). Phenotypic and genotypic characteristics of *Escherichia coli* strains of non-enteropathogenic *E. coli* (EPEC) serogroups that carry *eae* and lack the EPEC adherence factor and Shiga toxin DNA probe sequences. J. Infect. Dis. 183, 762–772. 10.1086/318821 11181153

[B56] Viljanen M. K. Peltola T. Junnila S. Y. Olkkonen L. Jarvinen H. Kuistila M. (1990). Outbreak of diarrhoea due to *Escherichia coli* O111:B4 in schoolchildren and adults: association of Vi antigen-like reactivity. Lancet 336, 831–834. 10.1016/0140-6736(90)92337-h 1976876

[B57] Vingadassalom D. Kazlauskas A. Skehan B. Cheng H. C. Magoun L. Robbins D. (2009). Insulin receptor tyrosine kinase substrate links the *E. coli* O157:H7 actin assembly effectors Tir and EspF_U_ during pedestal formation. Proc. Natl. Acad. Sci. U.S.A. 106, 6754–6759. 10.1073/pnas.0809131106 19366662 PMC2672544

[B58] Walters M. Sircili M. P. Sperandio V. (2006). AI-3 synthesis is not dependent on *luxS* in *Escherichia coli* . J. Bacteriol. 188, 5668–5681. 10.1128/JB.00648-06 16885435 PMC1540066

[B59] Wong A. R. Pearson J. S. Bright M. D. Munera D. Robinson K. S. Lee S. F. (2011). Enteropathogenic and enterohaemorrhagic *Escherichia coli*: even more subversive elements. Mol. Microbiol. 80, 1420–1438. 10.1111/j.1365-2958.2011.07661.x 21488979

[B60] Yamamoto D. Hernandes R. T. Blanco M. Greune L. Schmidt M. A. Carneiro S. M. (2009). Invasiveness as a putative additional virulence mechanism of some atypical Enteropathogenic *Escherichia coli* strains with different uncommon intimin types. BMC Microbiol. 9:146. 10.1186/1471-2180-9-146 19622141 PMC2724384

[B61] Yamamoto D. Hernandes R. T. Liberatore A. M. Abe C. M. Souza R. B. Romão F. T. (2017). *Escherichia albertii*, a novel human enteropathogen, colonizes rat enterocytes and translocates to extra-intestinal sites. PloS One 12, e0171385. 10.1371/journal.pone.0171385 28178312 PMC5298312

