# Supplementary figures and images for: Genomic Properties and Temporal Analysis of the Interaction of an Invasive Escherichia albertii With Epithelial Cells
Source: Front Cell Infect Microbiol. 2020 Dec 16;10:571088. doi: 10.3389/fcimb.2020.571088 (PMC7772469; doi:10.3389/fcimb.2020.571088)

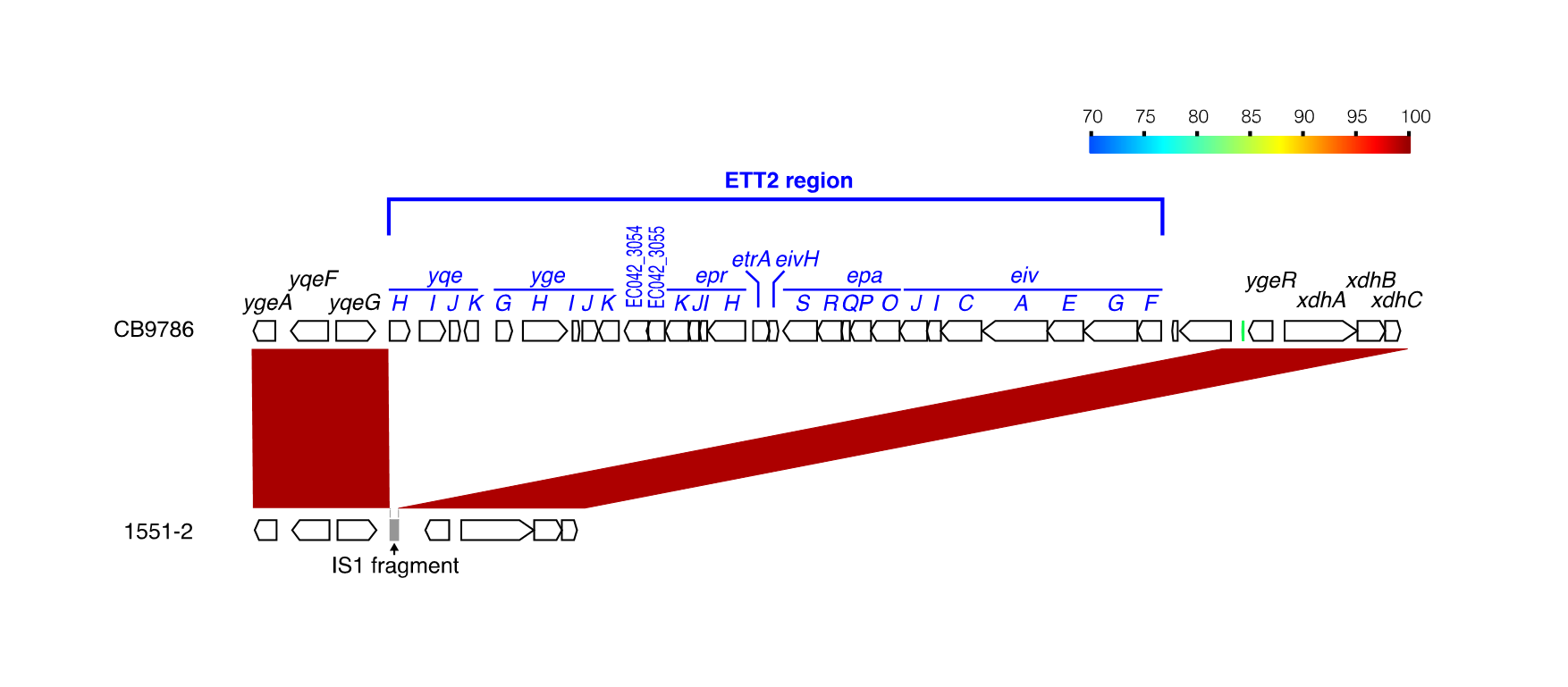

Supplement: Supplementary Figure 1 — Structural comparison of the ETT2 region of E. albertii CB9786 strain and the corresponding region of E. albertii 1551-2 strain. ETT2 was not detected in the 1551-2 strain. [file Image_1.tif]

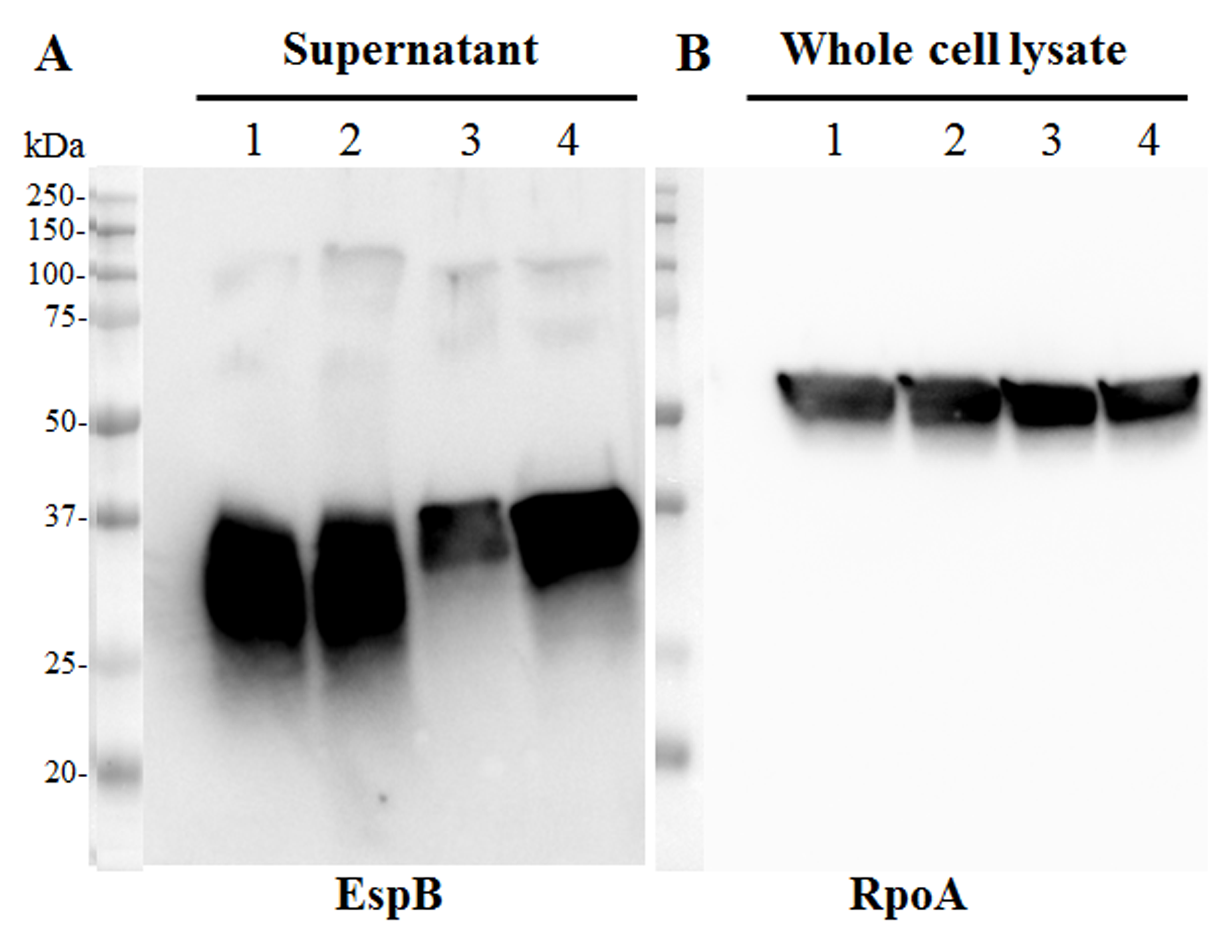

Supplement: Supplementary Figure 2 — Immunoblotting with anti-EspB (A) and anti-RpoA (B) antibodies, used as controls for the secreted protein preparation and bacterial cell lysate, respectively. Strains tested: E. albertii 1551-2 (lane 1), 1551-2 (pTccP3) (lane 2), EHEC 86-24 (lane 3), and EHEC (pKC471) (lane 4). The T3SS-dependent effector EspB and RpoA, the α subunit of the RNA polymerase core enzyme, were detected in all strains tested. [file Image_2.tif]

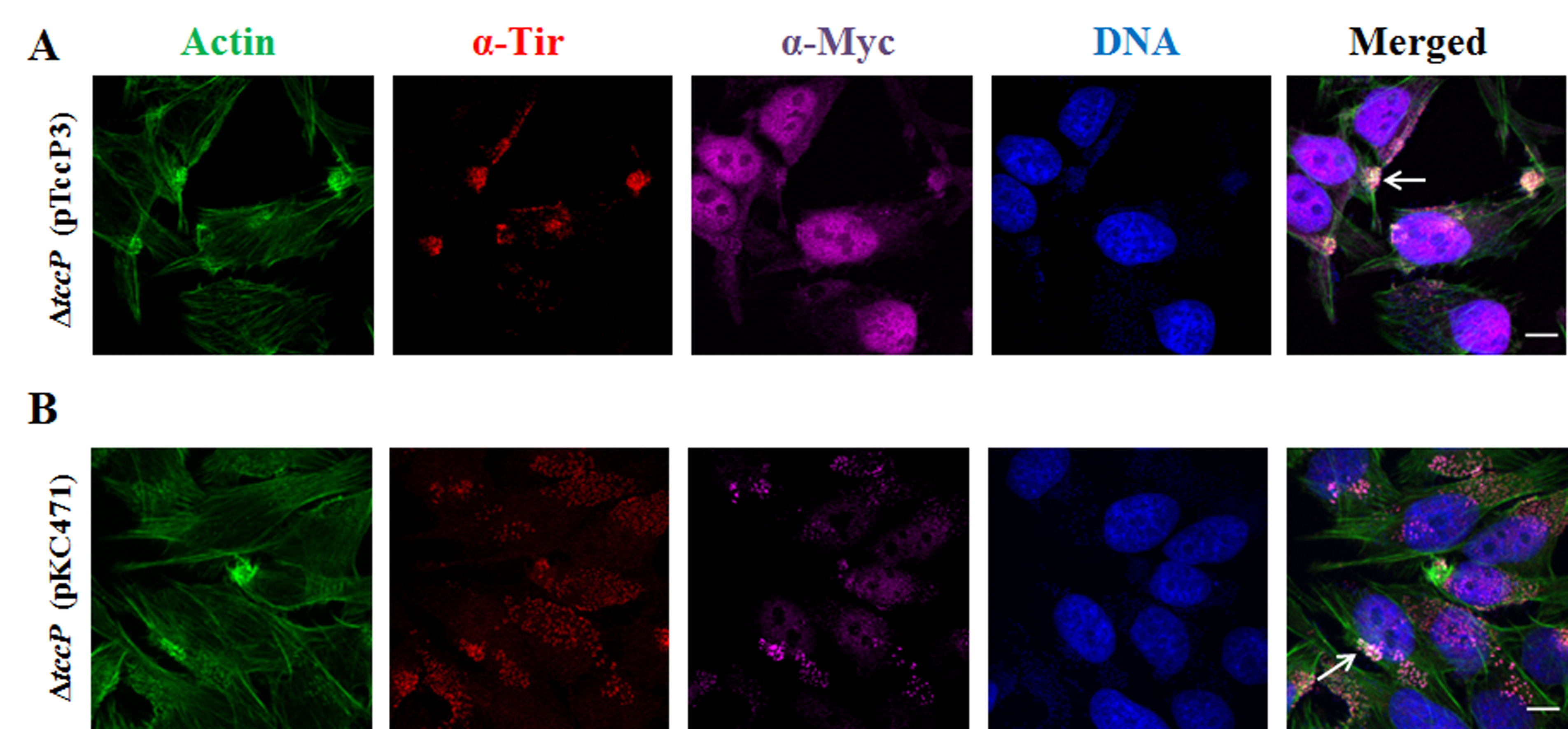

Supplement: Supplementary Figure 3 — Immunofluorescence showing the colocalization of polymerized F-actin with Tir and TccP3-Myc proteins. Note the colocalization (arrowheads) of polymerized F-actin with Tir and TccP3/TccP-Myc proteins in HeLa cells infected with EHEC 86-24ΔtccP strain harboring pTccP3 (A) or pKC471 (B) plasmids, respectively. Scale bar = 10 μm. [file Image_3.tif]

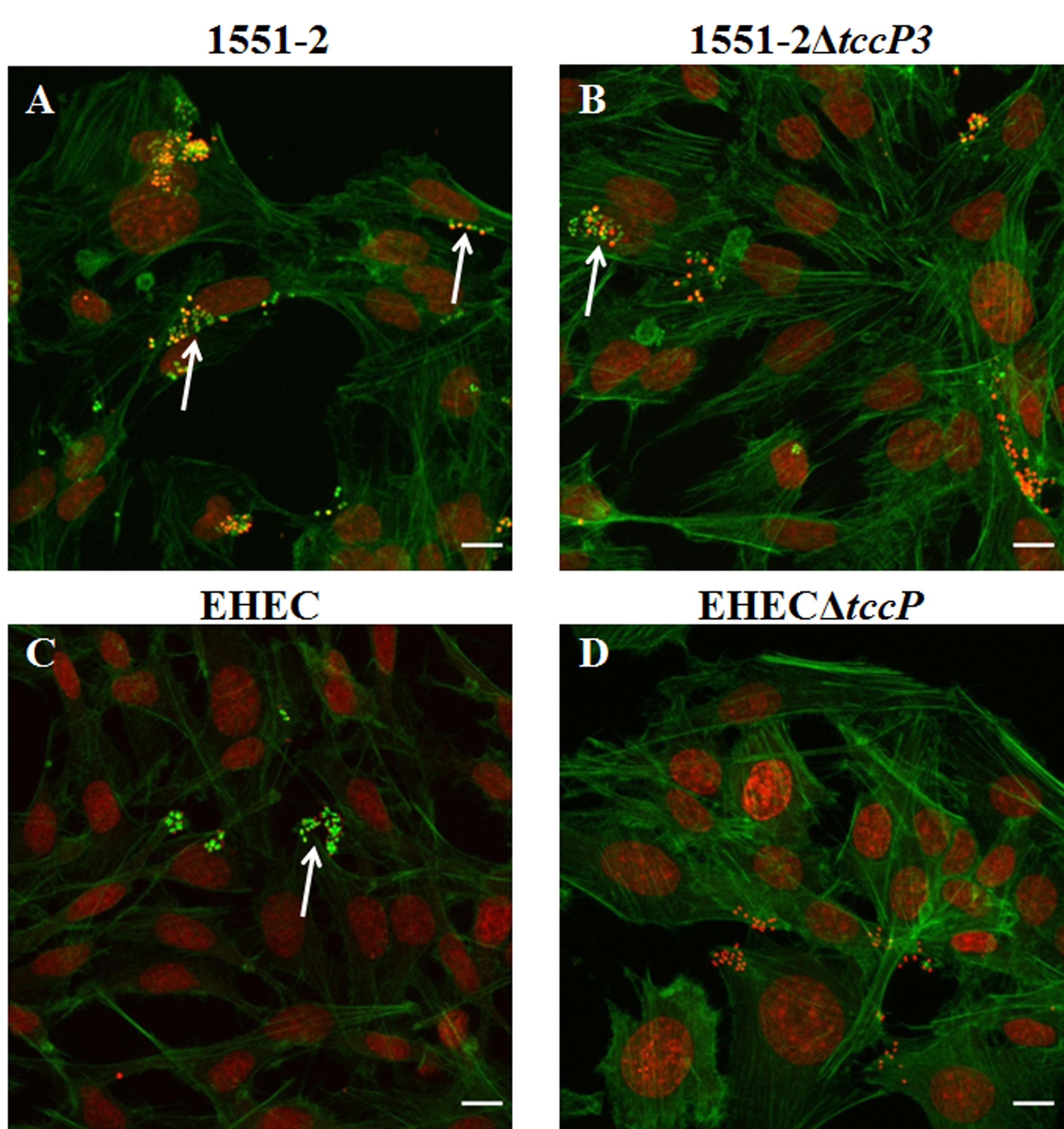

Supplement: Supplementary Figure 4 — Evaluation of the participation of TccP3 in F-actin polymerization in Nck-/- Mouse embryonic fibroblast (MEF) cells. The following strains were tested: E. albertii 1551-2 (A), 1551-2ΔtccP3 (B), EHEC 86-24 (C), and EHECΔtccP (D). Lack of TccP3 production did not affect the ability of the 1551-2ΔtccP3 mutant strain in triggering F-actin polymerization underneath adherent bacteria in an Nck-independent manner. Arrows indicate colocalization of adherent bacteria with polymerized F-actin. Scale bar = 10 μm. [file Image_4.tif]
